# Chemical Diversity of Three Endophytic *Talaromyces* Strains and Their Potential for Biocontrol against the Cocoa Pathogenic Fungus

**DOI:** 10.1002/cbdv.202503152

**Published:** 2026-01-10

**Authors:** Cecília L. S. Pereira, Gabrielle S. Paraguai, Thiago A. M. Brito, Josean F. Tavares, Sônia C. O. Melo, Eliane O. Silva

**Affiliations:** ^1^ Department of Organic Chemistry, Institute of Chemistry Universidade Federal da Bahia Salvador Brazil; ^2^ Departament of Biological Sciences Universidade Estadual de Santa Cruz Ilhéus Brazil; ^3^ Institute For Research in Pharmaceuticals and Medications Universidade Federal da Paraíba João Pessoa Brazil

**Keywords:** antifungal assay, biocontrol, metabolomics, *Moniliophthora perniciosa*, witches' broom disease of cacao

## Abstract

Biological control using beneficial endophytic fungi is a sustainable alternative to agrochemicals for managing plant diseases. This study assessed three endophytic *Talaromyces* strains, isolated from different Brazilian host plants, for antagonistic activity toward *Moniliophthora perniciosa*, the causative agent of cocoa witches’ broom disease. All strains inhibited the pathogen's mycelial growth *in vitro*, with *Talaromyces pinophilus* J6 exhibiting the strongest effect. Discriminant metabolites of the *T. pinophilus* J6 were unveiled by liquid chromatography‐tandem high‐resolution mass spectrometry‐based metabolomics combined with principal component analysis and a dereplication approach. These results underscore the promise of endophytic *Talaromyces* spp. as novel biocontrol agents and will contribute to ongoing research on sustainable agriculture. Moreover, chemical comprehension of endophytic fungi from the same genus that come from different host plants opens new insights into chemical ecology implicated in the endophyte‐plant interactions.

## Graphical abstract

Chemical profiling of endophytic Talaromyces strains reveals specialized metabolites with agricultural relevance, underscoring their potential application in the biocontrol of cocoa plant pathogens.

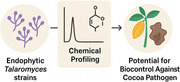

## 1 Introduction

*Theobroma cacao* L. (Sterculiaceae) is an economically important crop in several tropical countries, and its commercially valuable beans constitute about 10% of the cacao fruit's fresh weight [1]. Brazil is the seventh largest cocoa producer in the world and also the seventh largest exporter of the product and its derivatives. Between January and May 2022, 14 038 tons of chocolate, 20 232 tons of by‐products, and 273 tons of cocoa beans were exported by Brazil [2]. Fungal diseases are significant threats to economically important crops, leading to substantial losses due to their rapid spread and ability to adapt to different environmental conditions [3]. *Moniliophthora perniciosa* is a Basidiomycete that causes the witches' broom disease of cacao, being the cause of the rapid decline in cacao production in Brazil [4]. In 1989, witches’ broom disease became established in Bahia, a state in northeastern Brazil, and, together with other factors, it has contributed to the worsening impoverishment of the region, which continues to struggle to recover from its effects [5].

The use of chemical fungicides for the management of witches' broom disease has disadvantages such as the development of resistance by the plant pathogens, environmental contamination, and human health disturbances [6]. Alternative methods of controlling fungal crop diseases have been widely developed, including the use of disease‐resistant cultivars, adequate water and soil management, fertilization, crop rotations, and biological control agents, aiming to maintain or increase agricultural production with a reduced application of chemical agents [7]. Among the promising alternative control methods is the use of antagonistic microorganisms, such as beneficial endophytic fungi, capable of protecting the plant from the action of pathogens [8].

Endophytic microorganisms live inside plant tissues for at least part of their life cycles and are generally asymptomatic [9]. Within complex relationships, endophytic strains receive nutrients and protection, while plants have advantages, such as greater resistance in environments with intense stress caused by biotic (such as insects, herbivores, nematodes, and phytopathogenic microorganisms) or abiotic factors (such as pH, temperature, drought, and saline stresses) [10, 11]. Endophytic fungi inhabit a similar ecological niche to that occupied by phytopathogens, thus being able to protect their hosts and control pathogens through competition, production of antagonistic substances, direct parasitism, or even inducing resistance or tolerance [12]. Consequently, endophytes have attracted increasing attention as biological control agents and as inducers of plant defense responses [13, 14], which are key points for sustainable agriculture intensification. Their ability to synthesize a wide variety of specialized metabolites, such as alkaloids, terpenoids, polyketides, and peptides, underlies their potent antifungal, antibacterial, and insecticidal activities. Moreover, endophytic fungi enhance plant immunity through multiple mechanisms, including competitive exclusion, antimicrobial compound production, activation of defense pathways, phosphate solubilization, siderophore production, and modulation of phytohormones [15].

The endophyte microbiota composition significantly differs between distinct geographic areas [16]. Specific environmental characteristics act as “ecological filters” in the selection of the endophytic strains that are better adapted to local conditions. Additionally, some endophytes show host specificity [17] or are compatible with only specific host genotypes [18]. Within this context, the diversity of biomes and endemic plants found in Brazil represents a potential source of new beneficial microbial resources. Conducting studies to explore the biocontrol potential of endophyte biodiversity is a promising and powerful alternative to agrochemicals, offering control over a variety of plant fungal diseases. Fungi belonging to the *Talaromyces* genus have emerged as sustainable and effective agents for plant disease management. These organisms produce a broad range of bioactive metabolites exhibiting strong antifungal, antibacterial, and nematicidal activities, and secrete hydrolytic enzymes (such as chitinases, glucanases, and proteases) capable of degrading pathogen cell walls [19]. Moreover, their metabolites can induce systemic resistance and promote plant growth, enhancing overall crop vigor without reliance on synthetic stimulants. For instance, evidence from agricultural systems has demonstrated the efficacy of *Talaromyces purpurogenus* Q2 in enhancing the resistance of bitter gourd to fungal pathogens by activating the lignin biosynthesis pathway and simultaneously promoting plant growth [20].

**SCHEME 1:**
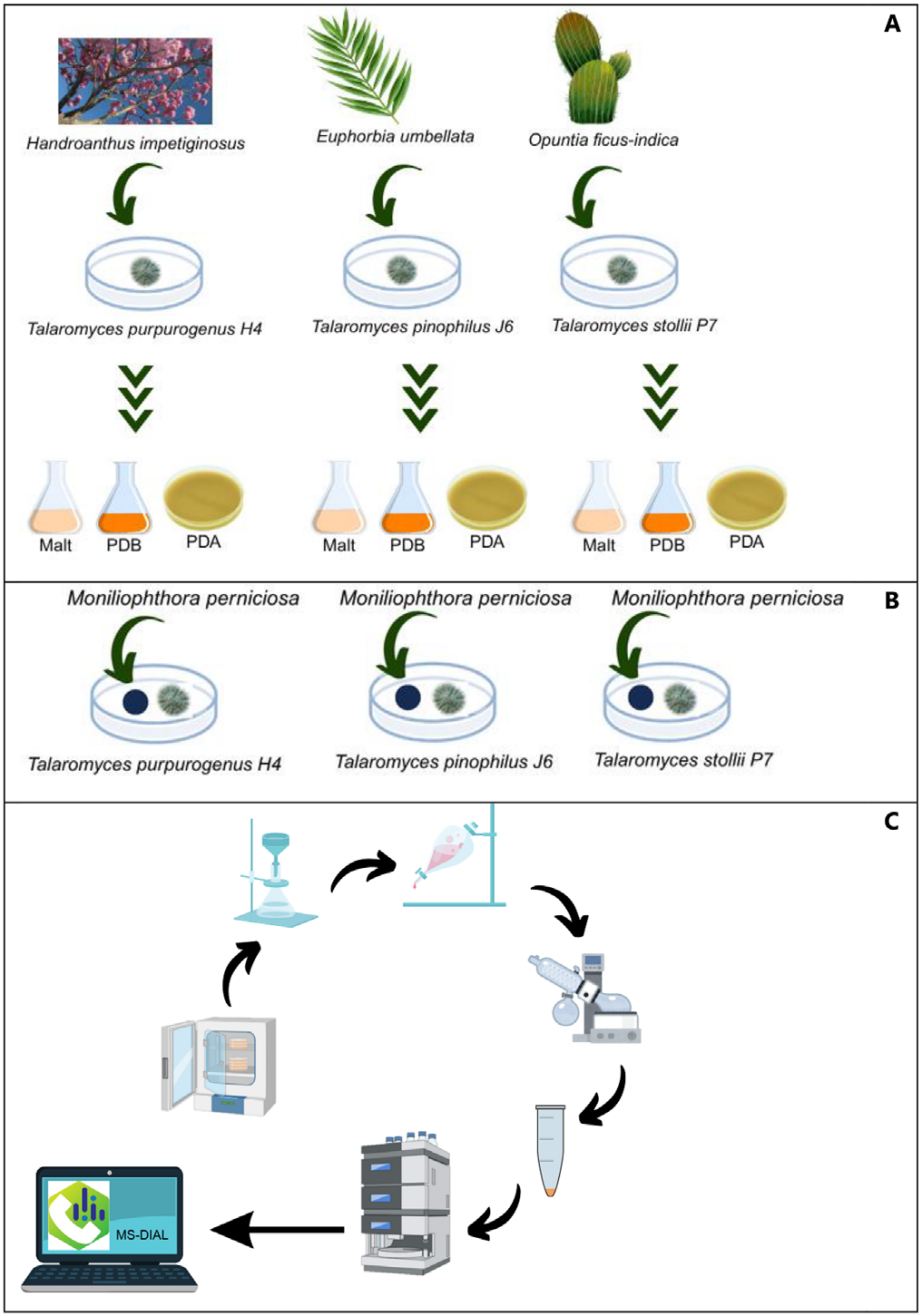
Schematic illustration of (A) Isolation of three endophytic *Talaromyces* strains from distinct Brazilian host plants, followed by their growth in different culture media; (B) Confrontation experiments between *Talaromyces* spp. and *Moniliophthora perniciosa*; and (C) Chemical comparison and metabolomic study of *Talaromyces* spp. crude extracts.

In this study, we investigated the antagonistic potential of three endophytic *Talaromyces* strains, each isolated from a distinct Brazilian host plant, against *M. perniciosa*, the causal agent of witches’ broom disease in cocoa (Scheme 1). Firstly, we qualitatively evaluated the interaction between the endophytes *Talaromyces* spp. and the pathogen by in vitro antagonism bioassays. To elucidate the chemical basis underlying their activity, we employed liquid chromatography‐high‐resolution mass spectrometry (LC‐HRMS)‐based metabolomics combined with principal component analysis and a dereplication approach to profile and compare their specialized metabolites. By linking chemical diversity to biocontrol performance, our results not only identify promising fungal candidates for sustainable cocoa disease management but also advances the understanding of the chemical ecology in endophyte–plant–pathogen interactions.

## 2 Results and Discussion

### 2.1 Endophytic Strains Identification

Three endophytic fungal strains—designated H4, J6, and P7 ‐ were isolated from the aerial parts of *Handroanthus impetiginosus*, *Euphorbia umbellata*, and *Opuntia ficus‐indica*, respectively. All colonies exhibited a consistent phenotypic progression, characterized by an initial yellow pigmentation that gradually shifted to green within seven days (Figure S1). Such reproducibility across isolates may reflect underlying metabolic or developmental similarities worthy of future investigation. Notably, this pattern was observed in the isolates H4, J6, and P7, which were obtained from taxonomically and ecologically distinct host plants, prompting further interest in molecular identification, comparative metabolomic profiling, and evaluation of their biological potential.

Genetic characterization of the endophytes H4, J6, and P7 elucidated the identity of all these strains as belonging to the *Talaromyces* genus (see phylogenetic trees in Figure S2A–C). The endophyte H4 clustered with sequences of *Talaromyces* spp. (83% similarity to reference sequences from this genus), while the strains J6 and P7 shared 99% and 100% identity, respectively, with the type strains *Talaromyces pinophilus* CBS 631.66 (JX091381) and *Talaromyces stollii* CBS 408.93 (JX315633). *Talaromyces* species have been frequently reported as endophytes in various plant hosts, reflecting their ecological versatility and adaptability to distinct environmental niches [21]. The observation that isolates H4, J6, and P7 originated from taxonomically unrelated host species raises intriguing questions regarding host specificity, ecological plasticity, and the conservation of biosynthetic pathways that may facilitate adaptation to different plant environments within the genus *Talaromyces*. Comparative metabolomic studies could provide deeper insights into these ecological and evolutionary dynamics.

The scientific interest in metabolites of fungi belonging to the *Talaromyces* genus (Ascomycota) has shown a constant increment since they are regarded as important natural product resources for producing various small molecules with diverse chemical structures and biological activities [22]. In our study, the three evaluated host plants naturally occur in distinct environmental conditions, which likely influence their specialized metabolism and the composition of their associated endophytic microbiota. Based on this, we hypothesize that the geographic origin of each host plant may also impact the metabolic profiles of the isolated endophytes (from the same genus), potentially shaping their biosynthetic capabilities and biological activities.

### 2.2 Inhibition of the Crop Pathogenic Fungus by the Evaluated Endophytic Fungi

The antagonistic potentials of *Talaromyces* sp. H4, *T. pinophilus* J6, and *T. stollii* P7 were separately evaluated against *M. perniciosa*, the causal agent of witches’ broom disease in cacao, using co‐culture assays for 7 and 14 days (Figure S3).

**FIGURE 1:**
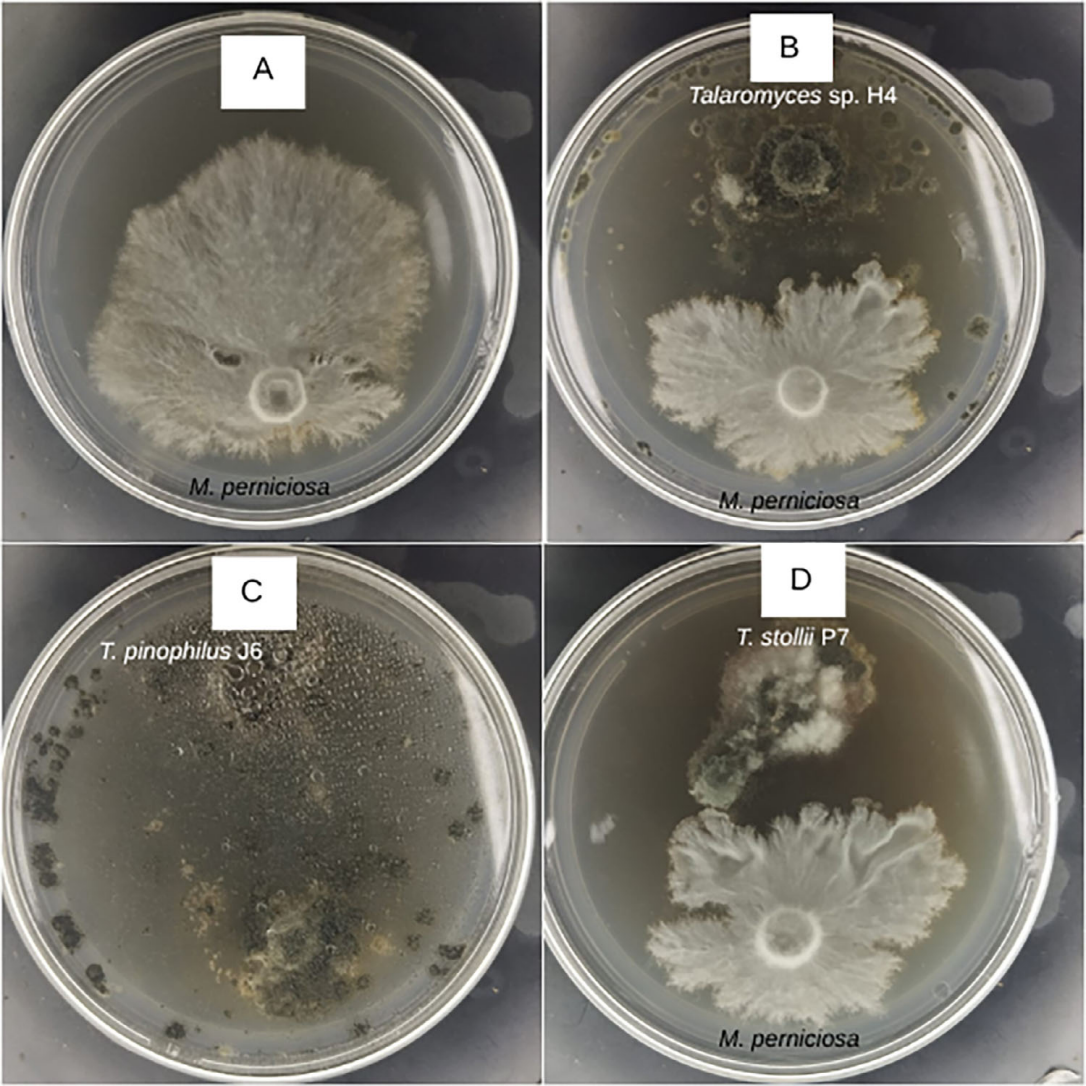
In vitro confrontation assays between *Moniliophthora perniciosa* and endophytic *Talaromyces* strains. Panels show: (A) *M. perniciosa* control, (B) *Talaromyces* sp. H4, (C) *Talaromyces pinophilus* J6, and (D) *Talaromyces stollii* P7. Each assay was performed on PDA plates under controlled conditions, and images were taken after 14 days to illustrate the inhibitory effect of each endophyte on the pathogen's mycelial growth. In the co‐culture assays (B–D), endophytic and pathogenic strains were inoculated in the upper and lower portions of the plate, respectively.

When compared with the control assay (*M. perniciosa*, Figure 1A), all *Talaromyces* spp. displayed inhibitory activity against *M. perniciosa* mycelial growth (Figure 1B–D), as the pathogen exhibited reduced growth in co‐culture with the endophytes relative to cultivation alone. *T. pinophilus* J6 (Figure 1C) displayed the strongest inhibitory effect, with *M. perniciosa* showing practically no mycelial development.

Inhibition outcomes were visually evaluated (for qualitative measurement) and categorized according to specialized literature as follows: (i) inhibition at a distance, when the opposing mycelia ceased growth before making contact; (ii) partial replacement, when the endophyte overgrew part of the pathogen colony without reaching its opposite edge; and (iii) complete replacement, when the endophyte entirely overgrew the pathogen colony. Accordingly, the interactions between *M. perniciosa* and *Talaromyces* sp. H4 (Figure 1B), *T. pinophilus* J6 (Figure 1C), and *T. stollii* P7 (Figure 1D) were classified as (i), (iii), and (i), respectively.

Microbial biological control agents have emerged as a viable alternative to chemical fungicides, playing a pivotal role in modern sustainable agriculture. Among these, endophytic fungi have garnered attention for their capacity to produce bioactive metabolites that suppress phytopathogens, promote plant growth, and enhance stress tolerance. Our preliminary inhibition findings reinforce the importance of chemically characterizing the endophytic *Talaromyces* spp., as such analyses could elucidate the distinct bioactivities observed against *M. perniciosa*.

### 2.3 Untargeted Metabolomics Analysis

Chemical investigations aimed at differentiating the metabolic profiles of the evaluated endophytes began with culturing the three *Talaromyces* strains on solid potato dextrose agar (PDA), liquid potato dextrose broth (PDB), and malt broth media. Liquid media were incubated under agitation and static conditions.

Base peak chromatograms (BPCs) of the ethyl acetate extracts from the controls (culture media without fungi, Figures S4) and the *Talaromyces* strains J6, H4, and P7 (Figures S5–S7, respectively) provide a visual overview of the chemical complexity of the samples and support the subsequent feature extraction and multivariate analyses. The BPCs exhibited stable baselines and well‐resolved peaks, confirming the high quality of the LC–HRMS acquisition. The positive ionization mode yielded superior signal intensity and a greater number of detected features; therefore, it was selected for the metabolomic analyses. The comparative analysis of BPCs revealed that cultures grown in Malt, PDA, and PDB media produced distinct chemical profiles across all evaluated fungal strains. Notably, agitation conditions induced clear differences in the chemical patterns of cultures in Malt medium compared with static conditions. In contrast, for cultures grown in PDB medium, the chemical profiles were relatively similar under both static and agitated conditions.

**FIGURE 2:**
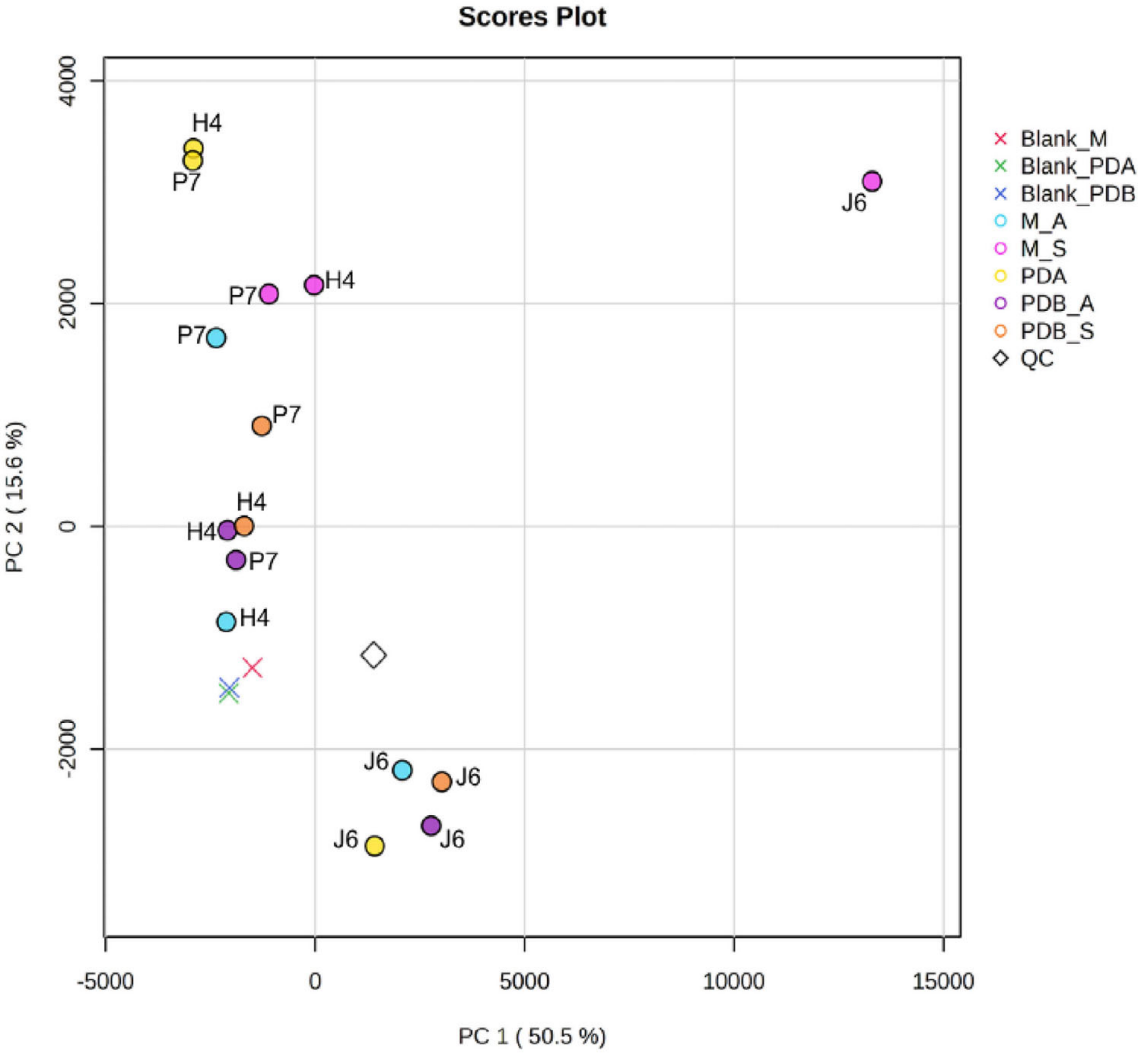
Scores plots of principal component analysis (PCA, PC1 vs. PC2 from five components) of metabolic fingerprints of *Talaromyces* spp. cultures generated using MetaboAnalyst, where Blank_M = malt medium control, Blank_PDA = Potato Dextrose Agar medium control, Blank_PDB = Potato Dextrose Broth medium control, M_A = malt medium with incubation under agitation, M_S = malt medium under static incubation, PDA = PDA medium, PDB_A = PDB medium with incubation under agitation, PDB_S = PDB medium under static incubation, QC = Quality Control.

Based on retention time and exact mass (*m*/*z*), the spot ID for each feature was analyzed using MS‐DIAL. Subsequently, the metabolic profiles of the ethyl acetate extracts from all *Talaromyces* spp. were explored through principal component analysis (PCA, Figure 2) and Hierarchical Clustering Analysis shown as a dendrogram (Figure S8). The PCA showed that 66.1% of the total variation in the data was represented by the first two principal components. Clustering analysis based on metabolomic similarity revealed a clear separation of *T. pinophilus* J6 from *Talaromyces* sp. H4 and *T. stollii* P7, regardless of the culture conditions. Thus, with negative scores for PC2, *T. pinophilus* J6 grown in PDA, PDB (under agitation or static conditions), and Malt (under agitation) were joined. Interestingly, the extract from *T. pinophilus* J6 grown in Malt medium incubated under static conditions was placed separately from all samples. In other words, among all assayed *Talaromyces* spp., the *T. pinophilus* J6 was chemically distinct (confirmed by Hierarchical Clustering Dendrogram), which may be correlated with its superior inhibition potential against *M. perniciosa* (Figure 1C).

### 2.4 Chemical Characterization of *Talaromyces* Strains

**FIGURE 3:**
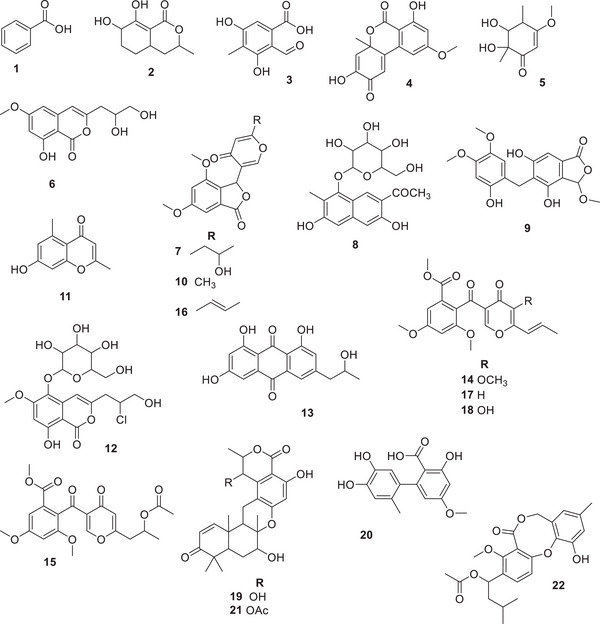
Chemical structures of specialized metabolites putatively identified in the ethyl acetate extracts of *Talaromyces* strains H4, J6, and P7 (the relative amounts are detailed in Figure S9). Metabolites were annotated based on liquid chromatography‐high‐resolution mass spectrometry (LC‐HRMS) accurate mass using an in‐house database. Structures are shown without stereochemical assignments when the absolute configuration could not be determined.

At the sequence, the three *Talaromyces* spp. were chemically characterized according to the tandem MS (MS/MS) fragmentation patterns, molecular formula, and in‐house database search, which includes over 700 specialized metabolites previously reported to *Talaromyces* sp. Twenty‐two metabolites were putatively identified (Table 1), and their chemical structures are depicted in Figure 3.

**TABLE 1:** Specialized metabolites putatively annotated in the ethyl acetate extracts of endophytic *Talaromyces* strains H4, J6, and P7, based on ultra‐high‐performance liquid chromatography‐electrospray ionization‐Quadrupole time‐of‐flight‐ mass spectrometry (UHPLC‐ESI‐QTOF‐MS) analysis and dereplication using a curated in‐house database. The table includes retention times (RT), observed *m/z* values, calculated errors (ppm), compound names, molecular formulas, and the *Talaromyces* strains in which each metabolite was previously reported.

No.	Retention time (min)	*m*/*z*, Adduct	*m*/*z* error (ppm)	Putative identification (Molecular formula)	Reported *Talaromyces* source (Reference)
1	17.52	140.0698, [M+NH_4_]^+^	−5.72	benzoic acid ^[a]^	*T. pinophilus* AF‐02
				(C_7_H_6_O_2_)	[23]
2	19.67	216.1217, [M+NH_4_]^+^	−6.02	(3*S*,4a*R*,7*S*)‐7,8‐dihydroxy‐3‐ methyl‐3,4,10,5,6,7‐hexahydro‐1*H*‐isochromen‐1‐one ^[b]^	*Talaromyces* sp.
				(C_10_H_14_O_4_)	[24]
3	25.02	179.0329, [M+H‐H_2_O]^+^	−5.59	2‐formyl‐3,5‐dihydroxy‐4‐methylbenzoic acid ^[c]^	*Talaromyces* sp. T1BF
				(C_9_H_8_O_5_)	[25]
4	27.19	311.0514, [M+Na]^+^	−3.86	dehydroaltenusin ^[c]^	*T. flavus*
				(C_15_H_12_O_6_)	[26]
5	27.85	209.0794, [M+Na]^+^	4.79	talaketide ^[b]^	*Talaromyces* sp. CY‐3
				(C_9_H_14_O_4_)	[27]
6	28.31	289.0695, [M+Na]^+^	4.15	diaportinol ^[c]^	*T. minnesotensis* BTBU20220184
				(C_13_H_14_O_6_)	[28]
7	32.85	347.1110, [M+H]^+^	−4.33	hydroxydihydrovermistatin ^[d]^	*T. thailandiasis*
				(C_18_H_18_O_7_)	[29]
8	33.26	377.1213, [M+H‐H_2_O]^+^	−4.77	talaminaphtholglycoside ^[d]^	*T. minnesotensis* BTBU20220184
				(C_19_H_22_O_9_)	[28]
9	33.57	345.0952, [M+H‐H_2_O]^+^	−3.48	talaromycolide A ^[d]^	*T. pinophilus* AF‐02.
				(C_18_H_18_O_8_)	[23]
10	34.21	303.0849, [M+H]^+^	−4.62	penisimplicissin ^[c]^	*T. thailandiasis*
				(C_16_H_14_O_6_)	[29]
11	34.23	191.0692, [M+H]^+^	−5.76	7‐hydroxy‐2,5‐dimethylchromon ^[c]^	*T. flavus*
				(C_11_H_10_O_3_)	[30]
12	35.94	485.0818, [M+Na]^+^	−0.62	talamiisocoumaringlycoside B ^[b]^	*T. minnesotensis* BTBU20220184
				(C_19_H_23_ClO_11_)	[28]
13	36.27	337.0669, [M+Na]^+^	−3.26	isorhodoptilometrin ^[c]^	*Talaromyces* sp.
				(C_17_H_14_O_6_)	[31]
14	37.54	411.1027, [M+Na]^+^	−5.59	3‐*O*‐methylfunicone ^[c]^	*T. pinophilus*
				(C_20_H_20_O_8_)	[32]
15	38.54	419.1314, [M+H]^+^	−5.49	actofunicone ^[b]^	*T. flavus* FKI‐0076
				(C_21_H_22_O_9_)	[25]
16	40.82	351.0824, [M+Na]^+^	−4.27	vermistatin ^[c]^	*T. thailandiasis*
				(C_18_H_16_O_6_)	[29]
17	43.02	359.1112, [M+H]^+^	−3.62	deoxyfunicone ^[d]^	*T. flavus* FKI‐0076
				(C_19_H_18_O_7_)	[25]
18	43.36	357.0960, [M+H‐H_2_O]^+^	−2.53	funicone ^[c]^	*T. flaus* IFM52668.
				(C_19_H_18_O_8_)	[25]
19	45.15	465.1867, [M+Na]^+^	−3.66	chrodrimanin A ^[a]^	*T. thailandensis* PSUSPSF059
				(C_25_H_30_O_7_)	[33]
20	45.52	313.0671, [M+Na]^+^	−3.84	biphenyl derivative ^[b]^	*T. flavus*
				(C_15_H_14_O_6_)	[30]
21	46.41	507.1968, [M+Na]^+^	−4.14	chrodrimanin B ^[a]^	*T. thailandensis* PSUSPSF059
				(C_27_H_32_O_8_)	[33]
22	52.11	437.1553, [M+Na]^+^	−4.12	vermixocin B ^[d]^	*Talaromyces* sp. LF458
				(C_23_H_26_O_7_)	[34]

^[a]^ mainly detected in both *Talaromyces* sp. H4 and *T. stollii* P7 cultures.

^[b]^ detected in all *Talaromyces* spp. cultures.

^[c]^ mainly detected *T. pinophilus* J6 cultures.

^[d]^ exclusively detected *T. pinophilus* J6 cultures.

Besides the 22 annotated metabolites, the MS‐DIAL analysis detected an additional 22 features (after removing blank features) that could not be confidently annotated using our in‐house database. The features that were detected but not annotated (Table S1) are potential candidates for future biochemical and biocontrol studies.

Certain compounds were predominantly detected in both *Talaromyces* sp. H4 and *T. stollii* P7 cultures (designated with a superscript letter ‘a’ in Table 1), while others were mainly or exclusively associated with *T. pinophilus* J6 cultures (designated with superscript letters ‘c’ and ‘d’ in Table 1). Figure S9 shows the relative distribution of each 22 annotated metabolites across the evaluated *Talaromyces* spp. Some specialized metabolites are conserved across the *Talaromyces* genus (designated with a superscript letter ‘b’ in Table 1), indicating shared biosynthetic pathways.

The use of an in‐house library for dereplication significantly enhanced the confidence in metabolite identification. Benzoic acid (**1**, *m/z* 140.0698), chrodrimanin A and B (**19** and **21**, *m/z* 465.1867 and 507.1968, respectively) were mainly detected in both *Talaromyces* sp. H4 and *T. stollii* P7 cultures. On the other hand, some metabolites were mainly detected in *T. pinophilus* J6 cultures: 2‐formyl‐3,5‐dihydroxy‐4‐methylbenzoic acid (**3**, *m*/*z* 179.0329), dehydroaltenusin (**4**, *m*/*z* 311.0514), diaportinol (**6**, *m*/*z* 289.0695), penisimplicissin (**10**, *m*/*z* 303.0849), 7‐hydroxy‐2,5‐dimethylchromon (**11**, *m*/*z* 191.0692), isorhodoptilometrin (**13**, *m*/*z* 337.0669), 3‐*O*‐methylfunicone (**14**, *m*/*z* 411.1027), vermistatin (**16**, *m*/*z* 351.0824), and funcione (**18**, *m*/*z* 357.0960). Other ones have exclusively been detected in the *T. pinophilus* J6 cultures: hydroxydihydrovermistatin (**7**, *m*/*z* 347.1110), talaminaphtholglycoside (**8**, *m*/*z* 377.1213), talaromycolide A (**9**, *m*/*z* 345.0964), deoxyfunicone (**17**, *m*/*z* 359.1112), and vermixocin B (**22**, *m*/*z* 437.1553).

The dereplication approach employed by us revealed a diverse array of specialized metabolites (Table 1). The presence of compounds with varied chemical scaffolds highlights their potential not only as biocontrol agents against plant pathogens but also as valuable resources for pharmaceutical and industrial biotechnological applications. Additionally, the chemical characterization of the *Talaromyces* strains evaluated herein showed some degree of specificity, which corroborates the chemical distribution pattern seen in PCA and HCA analysis. *Talaromyces* strains isolated from distinct host plants produce interesting repertoires of specialized metabolites. These chemical profiles appear to play a critical role in determining their antagonistic potential against *M. perniciosa*, the causal agent of witches’ broom disease. In particular, *T. pinophilus* J6 exhibited enhanced biocontrol activity, which correlates with the presence of bioactive compounds not detected in the other *Talaromyces* isolates.

The superior inhibitory activity displayed by *T. pinophilus* J6 against *M. perniciosa* (in comparison with the other two *Talaromyces* strains, Figure 1) could be due to the presence of chemicals with known antifungal activities. Funicone‐like compounds (**14**, **15**, **17**, and **18**) and phthalide derivatives, such as talaromycolide A (**9**) and vermistatin (**16**), have been reported as antimicrobial leads [23, 25, 29] and were mainly or exclusively detected in *T. pinophilus* J6 cultures.

Funicone‐like compounds have frequently been isolated from the *Talaromyces* genus and display a range of biological activities, such as fungicides, antivirals, and antitumorals [35]. Funicones and structurally related compounds represent a homogeneous group of fungal polyketides that were initially characterized as determinants of the antagonistic abilities by the producers against other microorganisms, but were later found to possess remarkable biological properties that have promoted their consideration as drug prospects [36]. Previously, 3‐*O*‐methylfunicone was tested on broad bean (*Vicia faba*) leaves against the pea aphid *Acyrthosiphon pisum* and displayed an interesting mortality [32]. It completely inhibited the growth of *Rhizoctonia solani* and other species of phytopathogenic fungi at a concentration of 0.1 mg/mL [37]. Funicone‐like compounds have been found exclusively or almost exclusively in *Talaromyces* strains and can be considered as candidates for the assessment of phylogenetic relations [38].

## 3 Conclusions

In summary, our results implied the potential of the endophytic *Talaromyces* spp. for further exploring their molecular diversity of natural products under more diverse culture conditions and discovering new bioactive compounds. Moreover, the chemical comparison of *Talaromyces* strains isolated from distinct host plants enriches our understanding of endophyte–host interactions and their influence on specialized metabolism. We demonstrated that endophytes from the same genus, isolated from distinct host plants, exhibited different chemical metabolism profiles, which were implicated in inhibitory activity against the phytopathogenic fungus at varying levels. Altogether, this study contributes valuable insights into the chemical ecology of endophytic fungi and lays the groundwork for future exploration of their bioactive compounds in agricultural and pharmaceutical contexts.

## 4 Experimental

### 4.1 Endophytic Fungi Isolation and Identification

The endophytic fungi coded as H4, J6, and P7 were isolated, respectively, from aerial parts of *Handroanthus impetiginosus* (Mart. ex DC.) Mattos, *Euphorbia umbellata* (Pax) Bruyns, and *Opuntia ficus‐indica* L. Mill, as previously reported by our research group [39, 40].

Host plants were collected from their characteristic Brazilian biomes: *H. impetiginosus* from the Cerrado biome in Minas Gerais State (S 21° 18′ 49.15″, W 45° 57′ 28.53″), and *E. umbellata* from the Atlantic Forest‐Cerrado transition area in Bahia State (S 14°54’46.81’, W 40°48’02.33’). In contrast, *O. ficus‐indica* was collected from the Caatinga biome, also in Bahia State (S 12° 59' 21.06 W 38° 30' 56.30). All samples were collected during the dry season. Only healthy, asymptomatic leaves were selected from plants in the vegetative phase, without visible flowers or fruits. *E. umbellata* and *O. ficus‐indica* were identified by Dr. Nádia Roque (Institute of Biology, Federal University of Bahia). A voucher specimen for each species was deposited in the Alexandre Leal Costa Herbarium (Federal University of Bahia) under the code ALBC 136527. *H. impetiginosus* was identified by Dr. Lúcia G. Lohmann (Department of Botany, Institute of Biosciences, University of São Paulo), and its voucher specimen was deposited in the Herbarium of the Federal University of Alfenas under the code 2535. The study was registered in the Brazilian System for the Management of Genetic Heritage and Associated Traditional Knowledge (SisGen) under the codes AE18457, AEFEF8D, and A30225F.

All strains were submitted to DNA sequencing and phylogenetic analysis for their identification. For this, the 7‐days cultures of the endophytic fungi were submitted to genomic DNA extraction and purification according to a previously reported protocol [41] by using glass microspheres (425–600 µm diameter, Sigma‐Aldrich).

ITS1–5.8S–ITS2 region from H4 was amplified using the primer pair ITS1–ITS4 [42]. The beta‐tubulin (*benA*) gene sequences were generated from J6 and P7 strains—Bt2a and Bt2b were the primers used. After purification (GFX PCR DNA and Gel Band Purification Kit; GE Healthcare, Little Chalfont, UK), the DNA fragments sequencing from each strain were carried out in an ABI 3500XL Series (Applied Biosystems, Foster City, USA) automatic sequencing. The DNA sequences were analyzed by Clustal X [43] with the MEGA 11.0 program [44]. Phylogenetic trees (Figure S1) were constructed using the Kimura model [45] and Neighbor‐Joining [46] parameter modeling (1000 bootstrap replications). The sequences data obtained from the endophytic fungi H4, J6, and P7 were registered at the GenBank database (https://www.ncbi.nlm.nih.gov/genbank/) with accession numbers MK749843, PQ963936, and PV755238, respectively.

### 4.2 Fungal Antagonism Assays

The endophytic fungal isolates H4, J6, and P7 were evaluated for antagonistic activity against the phytopathogen *M. perniciosa*. Dual‐culture assays were conducted in 9 cm Petri dishes containing PDA medium. Agar plugs (6 mm in diameter) from actively growing cultures (7 days old) of each endophytic strain and the pathogen were placed on opposite sides of the plate at the same time. The plates were incubated at 28°C under light conditions for 14 days, in triplicate. The inhibitions were evaluated after 7 and 14 days of the beginning of cultures.

The interaction patterns were categorized following established criteria [47]: (i) inhibition at a distance: the opposing mycelia stopped growing before contact; (ii) partial replacement: the endophyte overgrew part of the pathogen colony without reaching the opposite edge; (iii) complete replacement: the endophyte fully overgrew the pathogen colony.

### 4.3 Endophytic Fungi Cultures and Metabolites Extraction

The three endophytic fungi were grown in different culture media and conditions: (i) Petri dishes containing 20 mL of PDA, (ii) 250 mL‐Erlenmeyer flasks containing 100 mL of PDB, and (iii) 250 mL‐Erlenmeyer flasks containing 100 mL of malt extract medium (2.0% malt extract, 2.0% glucose, and 0.1% peptone). The liquid cultures were established with 10 plugs (6 mm diameter) of fungus, incubated under agitation (120 rpm), or under static conditions. All cultures were carried out in triplicate and incubated simultaneously for 15 days at 28°C. The crude extracts were obtained by extraction with ethyl acetate. The ethyl acetate extracts were concentrated using a rotary evaporator under reduced pressure at a temperature of 40°C to avoid thermal degradation of sensitive metabolites. After concentration, the residues were dried under a gentle stream of nitrogen to remove any remaining solvent before LC‐HRMS analysis. The blanks were achieved under the same conditions and consisted of the culture media without fungi.

### 4.4 Ultra‐HPLC‐HRMS Fungal Extracts Analysis

All crude extracts from *Talaromyces* cultures grown on Malt (shaken and static), PDB (shaken and static), and PDA were analyzed by ultra‐HPLC‐HRMS (UHPLC‐HRMS).

One microliter of each sample (0.5 mg/mL) was injected separately into the LC‐40D X3 (Shimadzu), equipped with the CBM‐40, DGU‐40S, LC 40D X3, SIL‐40C X3, and CTD‐40S modules, and coupled to an LCMS‐9050 mass spectrometer (Shimadzu) with an electrospray ionization (ESI) source and a Quadrupole Time‐of‐Flight (Q‐TOF) analyzer. The LC experiments were carried out using a C18 column (Kromasil‐250 mm × 4.6 mm x 5.0 µm). A linear gradient elution system was from 5% to 100% methanol (Supelco HPLC grade) in water for 60 min, under a flow rate of 0.6 mL/min and column temperature set to 40°C. The mass spectrometer's analysis parameters included a capillary voltage of 4.0 kV, a nebulizer gas (N_2_) flow rate of 3.0 L/min, a drying gas flow rate of 10 mL/min, and an interface temperature of 300°C.

The LC‐HRMS analyses were initially conducted in both positive and negative ionization modes. The positive ion mode provided higher signal intensity, greater feature richness, and better reproducibility for the ethyl acetate extracts of the *Talaromyces* strains. Consequently, we selected the positive ion mode (*m/z* 100–1200) for the metabolomic analyses presented in this study.

Fragmentation was performed by data‐independent acquisition (DIA), with an event time of 0.034 s, a loop time of 1.188 s, and a Q1 resolution of 34.4. Argon was the gas used in the collision cell, which operated at a collision energy of 30‐50. Product ions of *m/z* 50‐1200 were detected. The samples were randomly analyzed. The results from the sample replicates, quality control (QC) samples, and a blank analyzed at the beginning, middle, and end of the sequence were evaluated to ensure analytical consistency and instrument performance.

### 4.5 Mass Data Processing, Dereplication, and Metabolomics

To generate the metabolite annotation, the raw data from UHPLC‐MS obtained in positive ion mode were directly uploaded and deconvoluted using MS‐DIAL software (ver. 4.9).

MS‐DIAL is open‐source software that can be easily deployed in any laboratory environment with little or no programming required, primarily for analyzing and processing metabolomics data [48].

Data collection is performed with 0.01 Da MS1 tolerance and 0.05 Da MS2. Peak detection was applied with 5E5 amplitude for minimum peak height (threshold) and 0.1 Da mass slice width. Deconvolution parameters were set as follows: sigma window value of 0.5 and MS/MS abundance cutoff of 10 amplitudes. Alignment parameters setting included as reference file the QC sample (the most complex file), a retention time tolerance of 0.05 min. Finally, features based on blank information were removed.

All BPC data were inspected to verify chromatographic consistency and reproducibility across replicates before proceeding to feature extraction. After the processing and features generation, a dereplication methodology is used on an *in‐house* database.

Metabolite annotation was performed using a curated in‐house database comprising chemicals previously reported for the genus *Talaromyces*. This database includes molecular formulae, accurate masses, and MS/MS fragmentation patterns compiled from research articles and reviews (see references in Supporting Information). In total, our database contains 773 reported features. MS/MS data were acquired using DIA on a high‐resolution mass spectrometer in both positive and negative ion modes. The recorded fragmentation spectra were matched against the in‐house database using accurate mass (±6 ppm) and diagnostic fragment ions. Only tentative metabolite identifications were reported in this study.

The potential candidates for each metabolite were obtained by consulting the in‐house database. The dereplication was performed manually using our curated in‐house library of *Talaromyces* metabolites. Each detected feature was compared with reference data from the library based on exact mass and MS/MS fragmentation patterns. Although custom library integration could improve reproducibility, our in‐house spectral library was not MSP‐compliant for MS‐DIAL, which required manual dereplication.

To visualize sample grouping, an unsupervised multivariate data analysis was performed using MetaboAnalyst 6.0 software (https://www.metaboanalyst.ca). PCA analysis was used to explore the dataset with the capability to verify the linear/polynomial association among variable matrices by reducing the predictive model dimensions, allowing for simple discrimination among samples and the discrimination causing metabolite attributes.

## Author Contributions

**Cecília L. S. Pereira**: formal analysis, methodology. **Gabrielle S. Paraguai**: formal analysis and methodology – antagonism bioassays. **Thiago A. M. Brito**: formal analysis – mass data acquisition and analysis. **Josean F. Tavares**: funding acquisition, writing, review, and editing. **Sônia C. O. Melo**: formal analysis and methodology – antagonism bioassays. **Eliane O. Silva**: writing – original draft, project administration, supervision.

## Conflicts of Interest

The authors declare no conflicts of interest.

## Supporting information




**Supporting File 1**: cbdv70812‐sup‐0001‐SuppMat.docx.

**Supporitng File 2**: cbdv70812‐sup‐0002‐TableS1.xlsx.

## Data Availability

The authors have nothing to report.
