# Supplementary material for: Chemical Diversity of Three Endophytic Talaromyces Strains and Their Potential for Biocontrol against the Cocoa Pathogenic Fungus
Source: Chem Biodivers. 2026 Jan 10;23(1):e03152. doi: 10.1002/cbdv.202503152 (PMC12790416; doi:10.1002/cbdv.202503152)
Supplement: Supplementary file 1 — Supporting File 1: cbdv70812‐sup‐0001‐SuppMat.docx. [file CBDV-23-e03152-s001.docx]

Supplementary material

Chemical Diversity of Three Endophytic *Talaromyces* Strains and their Potential for Biocontrol Against the Cocoa Pathogenic Fungus

Cecília L. S. Pereira,^a^ Gabrielle S. Paraguai,^b^ Thiago A. M. Brito,^c^ Josean F. Tavares,^c^ Sônia C. O. Melo,^b^ and Eliane O. Silva^a*^

^a^Department of Organic Chemistry, Institute of Chemistry, Universidade Federal da Bahia, Salvador 40170-115, Bahia, Brazil (elianeos@ufba.br)

^b^Departament of Biological Sciences, Universidade Estadual de Santa Cruz, Ilhéus 45662900, Bahia, Brazil

^c^Institute for Research in Pharmaceuticals and Medications, Universidade Federal da Paraíba, Campus I, João Pessoa 58051900, Paraíba, Brazil

*Corresponding author:

elianeos@ufba.br (E.O. Silva)

Institute of Chemistry, Organic Chemistry Department, Federal University of Bahia (UFBA), Barão de Jeremoabo 147, 40170-115 Salvador, Bahia, Brazil


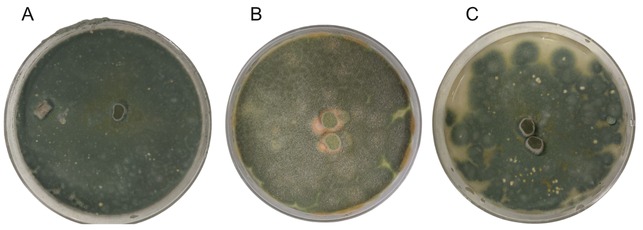


**Figure S1.** Axenic cultures of Talaromyces sp. H4 (A), Talaromyces pinophilus J6, and (B) Talaromyces stollii P7 (C) grown on PDA for 7 days.


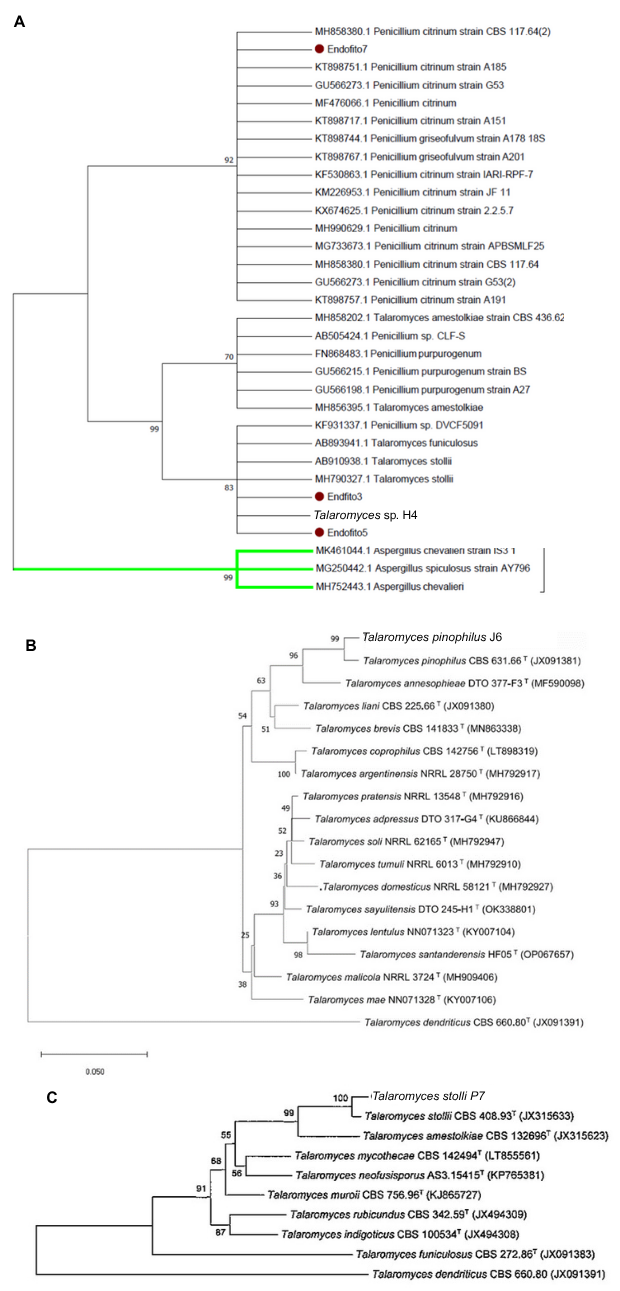


**Figure S2.** Phylogenetic positions based on ITS sequence of (**A**) *Talaromyces* sp*.* H4, and BenA sequences of (**B**) *Talaromyces pinophilus* J6 or (**C**) *Talaromyces stolli* P7. Phylogenetic trees were constructed by the Neighbor-Joining approach with MEGA 11.0. Bootstrap analysis was performed using 1,000 replications, and it is indicated at the nodes. The scale bars represent 0.050 substitutions per site.


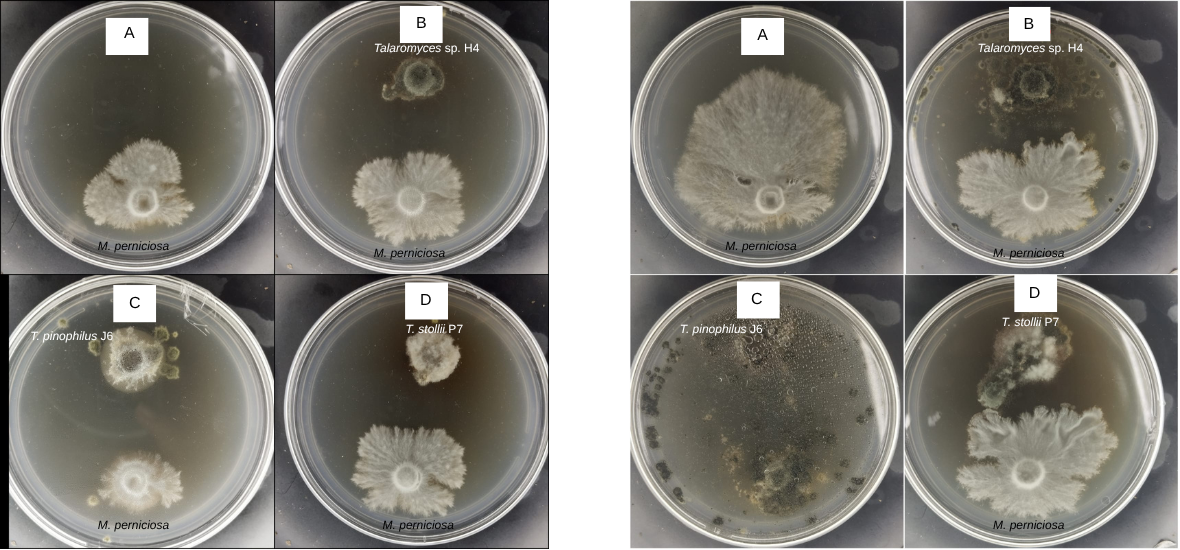


**Figure S3.** *In vitro* confrontation assays between Moniliophthora perniciosa and endophytic Talaromyces strains. Panels show: (A) M. perniciosa control, (B) Talaromyces sp. H4, (C) Talaromyces pinophilus J6, and (D) Talaromyces stollii P7. Each assay was performed on PDA plates under controlled conditions, and images were taken after 7 (left figure) or 14 (right figure) days to illustrate the inhibitory effect of each endophyte on the pathogen’s mycelial growth.

BLANK_M

BLANK_PDA

BLANK_PDB

QC

**Figure S4.** Base peak chromatograms (BPC) from UHPLC-HRMS (positive ion mode) of the ethyl acetate extract of controls: Blank_M = malt medium control, Blank_PDA = Potato Dextrose Agar medium control, Blank_PDB = Potato Dextrose Broth medium control, QC = Quality Control.

M_A_J6

M_S_J6

PDA_J6

PDB_A_J6

PDB_S_J6

**Figure S5.** Base peak chromatograms (BPC) from UHPLC-HRMS (positive ion mode) of the ethyl acetate extract of Talaromyces pinophilus J6 cultivated on M_A = malt medium with incubation under agitation, M_S = malt medium under static incubation, PDA = PDA medium, PDB_A = PDB medium with incubation under agitation, PDB_S = PDB medium under static incubation

M_A_H4

M_S_H4

PDA_H4

PDBD_A_H4

PDBD_S_H4

**Figure S6.** Base peak chromatograms (BPC) from UHPLC-HRMS (positive ion mode) of the ethyl acetate extract of Talaromyces sp. H4 cultivated on M_A = malt medium with incubation under agitation, M_S = malt medium under static incubation, PDA = PDA medium, PDB_A = PDB medium with incubation under agitation, PDB_S = PDB medium under static incubation

M_A_P7

M_S_P7

PDA_P7

PDB_A_P7

PDB_S_P7

**Figure S7.** Base peak chromatograms (BPC) from UHPLC-HRMS (positive ion mode) of the ethyl acetate extract of Talaromyces stollii P7 cultivated on M_A = malt medium with incubation under agitation, M_S = malt medium under static incubation, PDA = PDA medium, PDB_A = PDB medium with incubation under agitation, PDB_S = PDB medium under static incubation


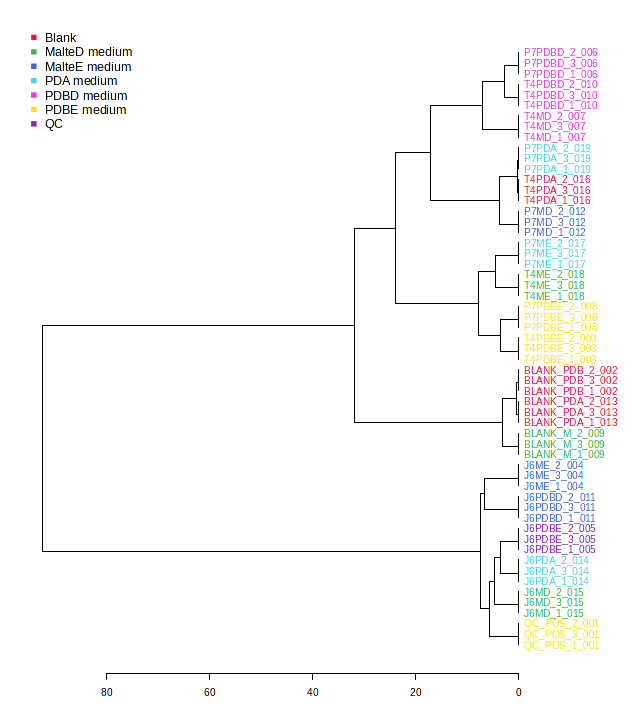


**Figure S8.** Hierarchical clustering analysis generated in MetaboAnalyst from metabolomic data, shown as a dendrogram, displaying sample grouping according to specialized metabolite similarities.

1. **
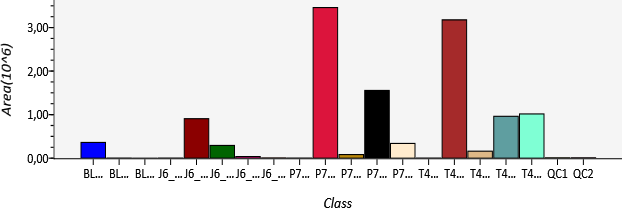
**
2. **
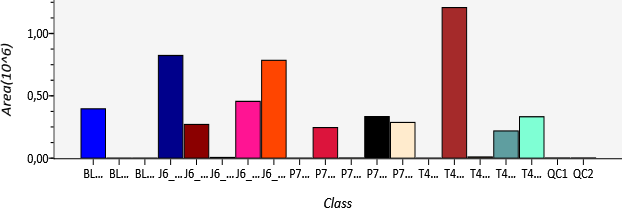
**
3. **
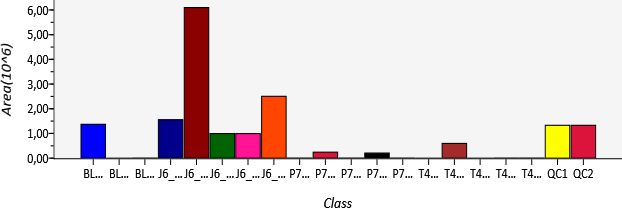
**
4. **
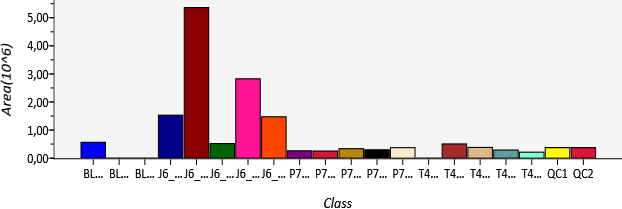
**
5. **
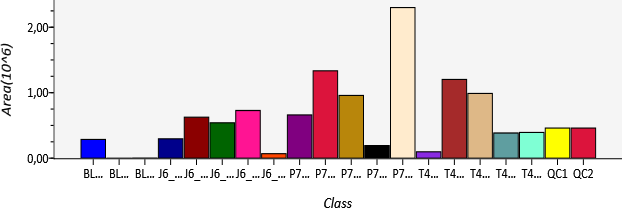
**
6. **
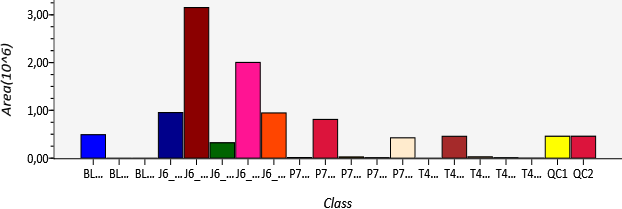
**
7. **
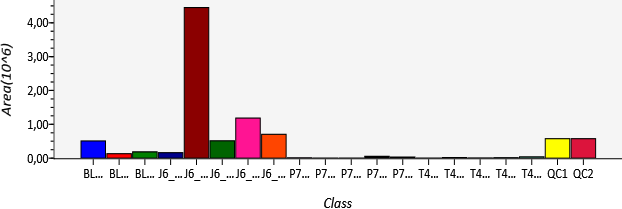
**
8. **
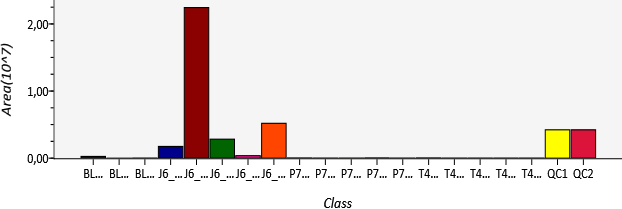
**
9. **
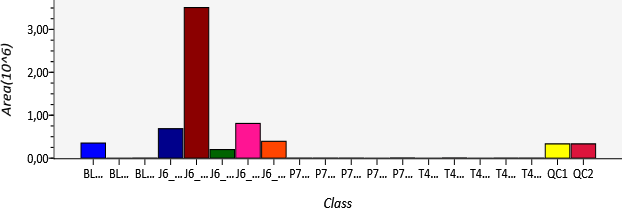
**
10. **
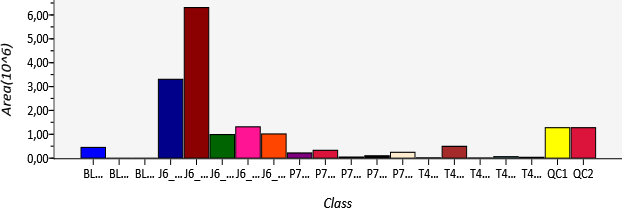
**
11. **
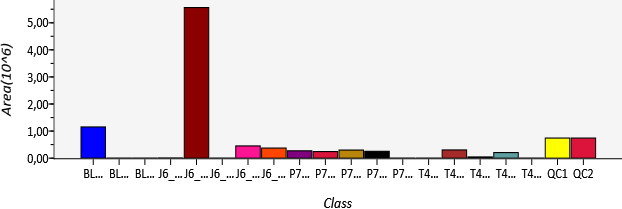
**
12. **
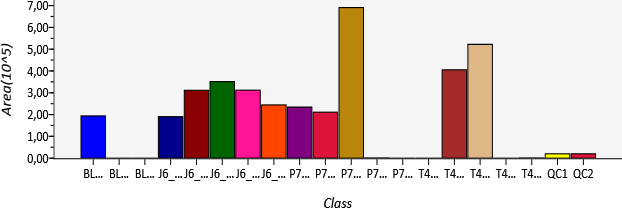
**
13. **
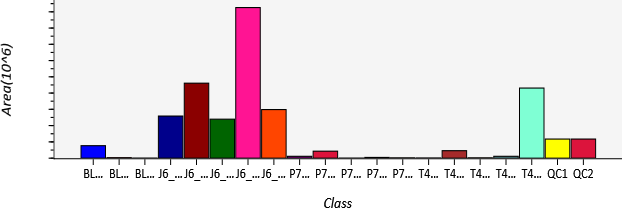
**
14. **
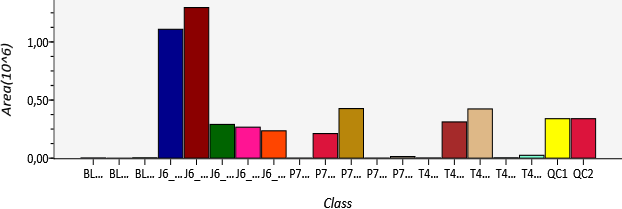
**
15. **
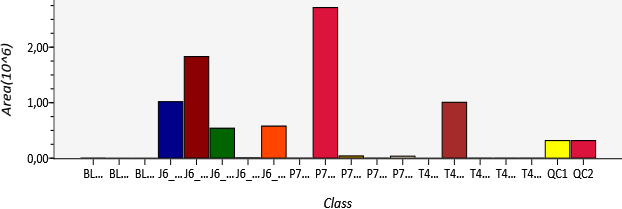
**
16. **
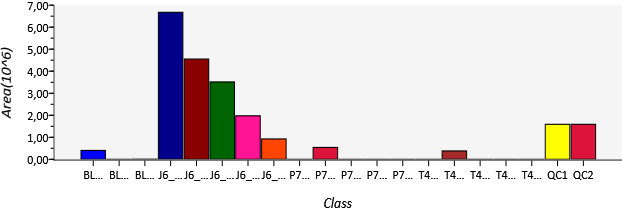
**
17. **
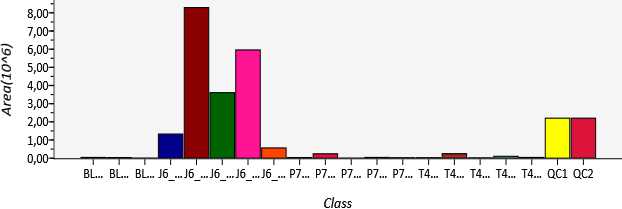
**
18. **
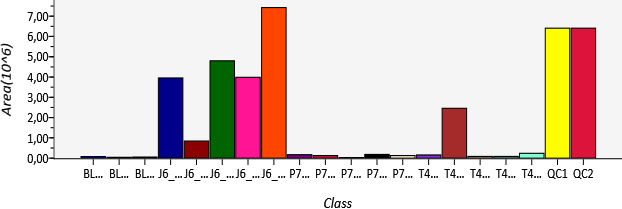
**
19. **
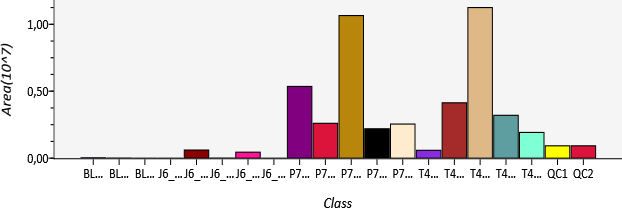
**
20. **
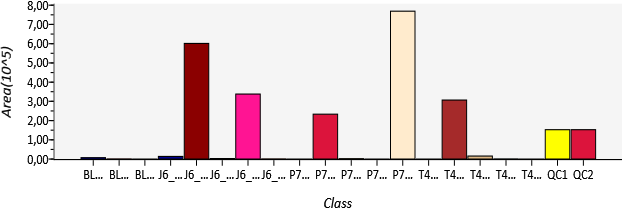
**
21. **
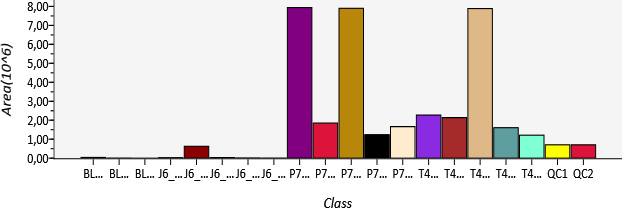
**
22. **
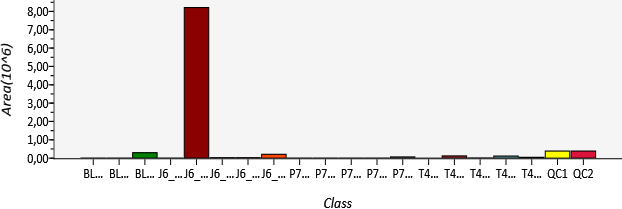
**

**Figure S9.** Chemical distribution of putatively identified specialized metabolites (compounds 1-22) among endophytic *Talaromyces* spp. The bar graphics were exported from MSDial software.

**Table S1:** The complete list of detected features, including mean values and standard deviations, can be found [here](all_features_triplicatas_mean_std.xlsx)

**In-house database**

Metabolite annotation was performed using a curated in-house database comprising chemical features previously reported for the genus Talaromyces. This database includes molecular formulae, accurate masses, and MS/MS fragmentation patterns compiled from research articles and reviews [1–29]

[1] M.-M. Zhai, J. Li, C.-X. Jiang, Y.-P. Shi, D.-L. Di, P. Crews, Q.-X. Wu, “The Bioactive Secondary Metabolites from Talaromyces species” *Nat Prod Bioprospect* 2016, *6*, 1–24.

[2] L.-R. Lei, L.-Q. Gong, M.-Y. Jin, R. Wang, R. Liu, J. Gao, M.-D. Liu, L. Huang, G.-Z. Wang, D. Wang, Y. Deng, “Research advances in the structures and biological activities of secondary metabolites from Talaromyces” *Front Microbiol* 2022, *13*, DOI 10.3389/fmicb.2022.984801.

[3] R. Nicoletti, R. Bellavita, A. Falanga, “The Outstanding Chemodiversity of Marine-Derived Talaromyces” *Biomolecules* 2023, *13*, 1021.

[4] G.-C. Fu, Z.-D. Yang, S.-Y. Zhou, X.-M. Li, H.-T. Yu, X.-J. Yao, J.-G. Fang, Z.-M. Shu, H.-Y. Xue, Y.-G. Wang, “Wortmannines A–C, three novel wortmannin derivatives with an unusual five-membered B ring from the endophytic fungus Talaromyces wortmannii LGT-4” *Tetrahedron Lett* 2016, *57*, 4608–4611.

[5] F. Vinale, R. Nicoletti, F. Borrelli, A. Mangoni, O. A. Parisi, R. Marra, N. Lombardi, F. Lacatena, L. Grauso, S. Finizio, M. Lorito, S. L. Woo, “Co-Culture of Plant Beneficial Microbes as Source of Bioactive Metabolites” *Sci Rep* 2017, *7*, 14330.

[6] M. Wang, L. Yang, L. Feng, F. Hu, F. Zhang, J. Ren, Y. Qiu, Z. Wang, “Verruculosins A–B, New Oligophenalenone Dimers from the Soft Coral-Derived Fungus Talaromyces verruculosus” *Mar Drugs* 2019, *17*, 516.

[7] F. Lacatena, R. Marra, P. Mazzei, A. Piccolo, M. C. Digilio, M. Giorgini, S. L. Woo, P. Cavallo, M. Lorito, F. Vinale, “Chlamyphilone, a Novel Pochonia chlamydosporia Metabolite with Insecticidal Activity” *Molecules* 2019, *24*, 750.

[8] T. O. Akinfala, J. Houbraken, M. Sulyok, A. R. Adedeji, A. C. Odebode, R. Krska, C. N. Ezekiel, “Moulds and their secondary metabolites associated with the fermentation and storage of two cocoa bean hybrids in Nigeria” *Int J Food Microbiol* 2020, *316*, 108490.

[9] L.-X. Feng, B.-Y. Zhang, H.-J. Zhu, L. Pan, F. Cao, “Bioactive Metabolites from Talaromyces purpureogenus, an Endophytic Fungus from Panax notoginseng” *Chem Nat Compd* 2020, *56*, 974–976.

[10] W. Yang, Q. Tan, Y. Yin, Y. Chen, Y. Zhang, J. Wu, L. Gao, B. Wang, Z. She, “Secondary Metabolites with α-Glucosidase Inhibitory Activity from Mangrove Endophytic Fungus Talaromyces sp. CY-3” *Mar Drugs* 2021, *19*, 492.

[11] S. P. Pelo, O. A. Adebo, E. Green, “Chemotaxonomic profiling of fungal endophytes of Solanum mauritianum (alien weed) using gas chromatography high resolution time-of-flight mass spectrometry (GC-HRTOF-MS)” *Metabolomics* 2021, *17*, 43.

[12] Z.-D. Yang, X.-D. Zhang, X. Yang, X.-J. Yao, Z.-M. Shu, “A norbisabolane and an arabitol benzoate from Talaromyces marneffei, an endophytic fungus of Epilobium angustifolium” *Fitoterapia* 2021, *153*, 104948.

[13] J. V. Christiansen, T. Isbrandt, C. Petersen, T. E. Sondergaard, M. R. Nielsen, T. B. Pedersen, J. L. Sørensen, T. O. Larsen, J. C. Frisvad, “Fungal quinones: diversity, producers, and applications of quinones from Aspergillus, Penicillium, Talaromyces, Fusarium, and Arthrinium” *Appl Microbiol Biotechnol* 2021, *105*, 8157–8193.

[14] X. Li, T. Awakawa, T. Mori, M. Ling, D. Hu, B. Wu, I. Abe, “Heterodimeric Non-heme Iron Enzymes in Fungal Meroterpenoid Biosynthesis” *J Am Chem Soc* 2021, *143*, 21425–21432.

[15] Y.-L. Li, J.-L. Yi, J. Cai, X.-M. Zhou, L. Chen, X. Zhuo, X.-Y. Lai, “Two new bioactive secondary metabolites from the endophytic fungus *Talaromyces assiutensis* JTY2” *Nat Prod Res* 2022, *36*, 3695–3700.

[16] K. Zhang, X. Zhang, R. Lin, H. Yang, F. Song, X. Xu, L. Wang, “New Secondary Metabolites from the Marine-Derived Fungus Talaromyces mangshanicus BTBU20211089” *Mar Drugs* 2022, *20*, 79.

[17] F. Song, Y. Dong, S. Wei, X. Zhang, K. Zhang, X. Xu, “New Antibacterial Secondary Metabolites from a Marine-Derived Talaromyces sp. Strain BTBU20213036” *Antibiotics* 2022, *11*, 222.

[18] A. J. al Fahad, “Putative Biosynthesis of Talarodioxadione &amp; Talarooxime from Talaromyces stipitatus” *Molecules* 2022, *27*, 4473.

[19] C. Cerracchio, V. Iovane, M. M. Salvatore, M. G. Amoroso, H. Dakroub, M. DellaGreca, R. Nicoletti, A. Andolfi, F. Fiorito, “Effectiveness of the Fungal Metabolite 3-O-Methylfunicone towards Canine Coronavirus in a Canine Fibrosarcoma Cell Line (A72)” *Antibiotics* 2022, *11*, 1594.

[20] Y. A. Hasanien, A. A. Nassrallah, A. G. Zaki, G. Abdelaziz, “Optimization, purification, and structure elucidation of anthraquinone pigment derivative from Talaromyces purpureogenus as a novel promising antioxidant, anticancer, and kidney radio-imaging agent” *J Biotechnol* 2022, *356*, 30–41.

[21] H. Farhat, F. Urooj, N. Sohail, S. F. Hameedi, M. S. Ali, S. Ehteshamul-Haque, “Evaluation of antibacterial potential of endophytic fungi and GC-MS profiling of metabolites from Talaromyces trachyspermus” *South African Journal of Botany* 2022, *150*, 240–247.

[22] I. A. Soliman, Y. A. Hasanien, A. G. Zaki, H. A. Shawky, A. A. Nassrallah, “Irradiation impact on biological activities of Anthraquinone pigment produced from Talaromyces purpureogenus and its evaluation, characterization and application in beef burger as natural preservative” *BMC Microbiol* 2022, *22*, 325.

[23] M. Rani, S. Jaglan, V. Beniwal, V. Chhokar, “Bioactive saponin profiling of endophytic fungi from *Asparagus racemosus*” *Nat Prod Res* 2023, *37*, 3889–3895.

[24] D. Zhang, X. Wang, B. Liu, S. Li, Y. Wang, T. Guo, Y. Sun, “New Dipyrroloquinones from a Plant-Derived Endophytic Fungus Talaromyces sp.” *Molecules* 2023, *28*, 7847.

[25] M. Dutta, A. Hazra, E. Bhattacharya, R. Bose, S. Mandal Biswas, “Characterization and metabolomic profiling of two pigment producing fungi from infected fruits of Indian Gooseberry” *Arch Microbiol* 2023, *205*, 141.

[26] Z.-D. Yang, J. Ma, Z.-J. Li, J. Jin, L. Wang, Z.-M. Shu, “Secondary Metabolites of the Endophytic Fungi Talaromyces flavus Cultivated in Pumpkin Medium and Their Bioactivity” *Chem Nat Compd* 2023, *59*, 549–552.

[27] W. Wang, J. Wang, F. Song, R. Jia, L. Wang, X. Xu, N. Yang, “New Secondary Metabolites from Marine-Derived Fungus Talaromyces minnesotensis BTBU20220184” *Mar Drugs* 2024, *22*, 237.

[28] B. N. S. Ningsih, V. Rukachaisirikul, S. Phongpaichit, C. Muanprasat, S. Preedanon, J. Sakayaroj, R. Intayot, S. Jungsuttiwong, “Talarostatin, a vermistatin derivative from the soil-derived fungus *Talaromyces thailandensis* PSU-SPSF059” *Nat Prod Res* 2024, *38*, 2535–2542.

[29] Y. Wang, X.-M. Li, N. Song, B.-G. Wang, H.-L. Li, L.-H. Meng, “Secondary metabolites with fungicide potentials from the deep-sea seamount-derived fungus Talaromyces scorteus AS-242” *Bioorg Chem* 2024, *147*, 107417.
